# Surface Chemistry of Gold Nanoparticles Modulates Cytokines and Nanomechanical Properties in Pancreatic Cancer Cell Lines: A Correlative Study

**DOI:** 10.26502/fjhs.170

**Published:** 2024-03-13

**Authors:** Tanmay Kulkarni, Ramcharan Singh Angom, Enfeng Wang, Debabrata Mukhopadhyay, Santanu Bhattacharya

**Affiliations:** 1 Department of Biochemistry and Molecular Biology, Mayo Clinic College of Medicines and Science, Jacksonville, FL, United States; 2 Department of Physiology and Biomedical Engineering, Mayo Clinic College of Medicines and Science, Jacksonville, FL, United States

**Keywords:** Endocytosis, Nanomechanical attributes, Chemokines, Interleukins, Gold nanoparticles, Pancreatic cancer, Pearson correlations

## Abstract

Surface chemistry of nanoparticles play significant role in their cellular interaction. Along with other group, we previously demonstrated that dynamic alteration of cell membrane during uptake of gold nanoparticles can be thoroughly probed by nanomechanical properties of cell membrane. Additionally, endocytosis influences intracellular cytokines expression that also impact membrane stiffness. Hence, we have hypothesized that surface chemistry of gold nanoparticles influences intracellular cytokines which in turn imparts dynamic alteration of nanomechanical properties of cellular membrane of pancreatic cancer cells. Various gold nanoparticles decorated with targeting peptide, polyethylene glycol or their combinations have been used to treat two pancreatic cancer cell lines, Panc-1 and AsPC1, for 1 and 24 hours. Atomic force microscope is used to measure linear and nonlinear nanomechanical properties of cell membrane. Intracellular cytokine has been measured using real time polymeric chain reaction. We evaluated several criteria such as receptor dependent vs independent, PEGylated vs non-PEGylated and different timepoints, to deduce correlations between cytokines and nanomechanical attributes. We have identified unique relationship pro-tumorigenic cytokines with both linear and non-linear nanomechanical properties of Panc-1 and AsPC1 cell membrane during uptake of pristine gold nanoparticles or for PEGylation and for targeting peptide conjugation at the nanoparticle surface.

## Introduction

Endocytosis is one of the fundamental processes involved in cellular communication with the surrounding environment [1, 2]. Mechanisms underlying in uptake of various therapeutic entities including drug conjugated gold nanoparticles (GNP) by the cells are extremely complex and yet to understand in detail [3–6]. Current literatures identified several factors such as physical, chemical and mechanical attributes of GNPs, as well as the cell types play a critical role in deciding the type of endocytosis [7–12]. One of the studies focused on the size dependency of GNPs on endocytosis and demonstrated that 45 nm sized GNPs were better for drug delivery compared to larger sized nanoparticles (for instance, 75 nm) in HeLa and small lung cancer cells [13]. Another study focused on noncircular shapes such as star, rods and triangles in RAW254.7 macrophage cells and observed that the efficiency of uptake from lowest to highest was as per the abovementioned order [14]. There have been several studies pertaining to endocytosis of GNPs in the past focusing mainly on the signaling mechanisms/events contributing to endocytosis [15–18]. A few studies have focused on the cell surface topography such as actin reorganization and pit formation during the uptake process [19–21]. Our prior work conclusively demonstrated a direct correlation between the plectin-1 receptor expression at the surface of pancreatic cancer cell lines and their membrane stiffness expressions [22, 23]. More recently, our group investigated the dynamic alteration in membrane nanomechanical properties (NMPs) and concluded that non-linear poroelastic attributes are more detrimental than their linear attributes counterparts during receptor dependent and independent endocytosis [22, 23]. Additionally, intracellular alterations of cytokines can regulate architecture of cell membranes that can influence membrane stiffness.

Cytokines are known to be involved in various endocytosis pathways [24–27]. For instance, Interleukin (IL-2R) involves activities of dynamin and actin-modulating GTPases Rac1 and RhoA for internalization [25]. Moreover, it was also demonstrated that IL-2R acts as cargo of fast endophilin-mediated endocytosis, where endophilins interact with dynamin and actin cytoskeleton [26, 27]. Another cytokine IL-7 signal transduction is necessary for clathrin-dependent endocytosis and that it triggers rapid IL-7Rα endocytosis, essential for IL-7 mediated signalling and subsequent receptor degradation [28]. Clathrin independent (also known as receptor independent) mechanism heavily relies on actin, dynamin and their partners such as Rac1 and P21 Activated kinase 1/2 [PAK1/2], whereas clathrin dependent (also known as receptor dependent) mechanisms rely on membrane receptors that bind to the ligands present on the cell surface and internalize [22, 23]. Both types of processes involve reorganization of actin during the internalization process leading to alteration in cellular NMPs [29, 30]. Hence, it is imperative to evaluate the alterations in cellular NMPs because of nanoparticles endocytosis and their correlation with cytokine alterations. To characterize nanomechanical attributes of various biological entities, atomic force microscopy (AFM) tool is commonly employed [31–33]. AFM allows fixation and stain free characterization of cellular membrane in fluid environment thus, preserving its integrity [34, 35]. It has been employed to distinguish cancer cells from normal cells [36–38] as well as in senescence paradigm [39, 40]. AFM has also been used to study the dynamic alteration in membrane linear attributes such as stiffness, deformation and adhesion [22, 41]. as well as non-linear attributes such as drained Poisson’s ratio, diffusion coefficient and pore size [23] during receptor dependent and independent processes. However, we don’t have adequate knowledge about the consequence of intracellular cytokine regulation and nanomechanical alteration of cellular membranes during endocytosis of GNPs with wide variety surface chemistry.

In this work, we hypothesize that certain pro-tumorigenic cytokines and NMPs are correlated on the type of endocytosis and surface chemistry of GNPs containing wide varieties of biologics. To test this hypothesis, we have employed two different pancreatic cancer cell lines namely Panc-1 and AsPC-1 and treated them with pristine GNP (p-GNP), PEGylated GNP (PEG-GNP), Plectin-1 targeted peptide conjugated with GNP (PTP-GNP) and PTP-GNP coated with PEG (PTP-GNP-PEG) for 1 hour (hr) and 24 hr time points. These treated cells were then evaluated under the AFM to understand NMPs of cell membranes. Additionally, we have identified several protumorigenic cytokines that have direct influence on the uptake of GNPs. The normalized values of cellular NMPs and cytokine expressions were then used to calculate correlations between them that gives insights into their relationships.

## Materials and Methods

### Cell culture

Human pancreatic cancer cell lines such as Panc-1 and AsPC-1 were purchased from American Type Culture Collection (ATCC; Manassas VA) and used with no further validations. Cells were seeded in a 60-mm dish at 40–50% confluency and allowed to culture at 37°C in a humidified incubator maintained at 5% CO_2_ atmosphere till the confluency was obtained at 70–80% for the AFM experiments and 90% for qRT-PCR experiments. Panc-1 and AsPC-1 cells were cultured using Dulbecco’s Modified Eagle Medium (DMEM) and Roswell Park Memorial Institute (RPMI) 1640 medium both purchased from Gibco (Billings MT). Both media were supplemented with 10% Heat-Inactivated Fetal Bovine Serum (HI-FBS) (purchased from Gemini Bio-Products, Sacramento CA) and 1% Penicillin Streptomycin purchased from Gibco.

### Gold nanoparticles synthesis

All the nanoformulations used in this work have been synthesized according to our previous work [42]. Briefly, Gold (III) chloride trihydrate and plectin-1 targeted peptide (KTLLPTPYC, purchased from Biomatik; Wilmington DE) was fluxed in Milli-Q water in 10:1 M ratio and stirred from 2 minutes (mins) at 37°C. Following which, 1 M sodium hydroxide (NaOH) was carefully added dropwise to accomplish an overall pH 12 solution. Post stirring the solution continuously for 16 hr, GNPs were isolated by ultra-centrifuge (purchased from Beckman Coulter Inc., Brea, CA). GNP pellet was separated from the supernatant and immediately added with Milli-Q water to make the ultimate volume exact same as the initial reaction volume. To PEGylate PTP-GNP, it was further diluted 10X and mixed with 2.5 mg/mL of mPEG_3_-SH for 30 mins at room temperature followed by subjecting the mixture to ultracentrifugation at 38K RPM. These nanoformulations were subjected to various characterizations described previously [22]. We observed a consistent size of ~5 nm measured using dynamic light scattering (DLS) and around 53 peptides available on the GNP surface obtained using computational modeling simulation described elsewhere previously [42].

### Real-Time Quantitative Reverse Transcription

To evaluate mRNA expression analysis of the cytokine expressions, total RNA was extracted using RNeasy Kit (purchased from Qiagen). Following which, 1 μg of total RNA was reverse transcribed using an iScript cDNA synthesis kit (purchased from Bio-Rad) according to the manufacturer’s protocol. PCR was then performed using 0.1 μg of cDNA in a 10 μL of PCR mix containing 500 nM of each primer and power Sybr master mix (purchased form Life Technologies). The reaction was performed using 7500 PCR system (purchased from Applied Biosystems) and the parameters applied were 40 cycles of amplification at 95 °C for 15 seconds and 57 °C for 1 min. The PCR primers and their sequences are mentioned in the supplementary table S1. These experiments were performed in triplicates.

### Atomic Force Microscopy

AFM experiments were performed using Dimension Fastscan with ScanAsyst incorporating ICON head scanner (Bruker Corp, Santa Barbara, CA) for linear and non-linear NMPs evaluation. We incorporated LC CAL A probes designed specifically for cell related studies. Nominal spring constant (K) and tip radius recommended by the manufacturer is 0.1 N/m and 70 nm, respectively. Laser alignments were performed to yield maximum signal strength. Foremost, the probe was calibrated on a blank petri dish in fluid medium to account for alterations in NMPs due to hydrodynamic drag resulting from the fluid medium. The calibration process concluded the spring constant to be 0.08 N/m and the deflection sensitivity to be 26.9 nm/V along with the amplitude sensitivity of 772 nm/V at 1 KHz tapping frequency. The peak force amplitude was set to 300 nm for accurate tip-sample interaction and protect tip from damage during initial approach towards the sample. AFM is a surface mapping tool; hence we restricted all the measurements over a 500 × 50 nm^2^ region over the nuclear membrane to overcome heterogeneity in its surface roughness owing to the presence of actin fibers, microtubules and bilipid layer according to our previous studies [22, 23, 39, 43]. We used a custom-built petri dish holder to hold the culture dish firmly during all the AFM experiments. Cells in the culture dish were identified using an optical microscope attached to the ICON head scanner. Further, we performed nanoindentation and force ramp script experiments to evaluate linear and non-linear NMPs, respectively. The ramping parameters were optimized according to our previous published studies [43]. Nanoindentation technique involves the approach of the tip followed by indenting into the sample driven by the applied force and finally, retracting from the sample, resulting into a force-separation (F-S) curve. The entire nanoindentation process at a single point occurs over a few milliseconds’ duration. For ramp script analysis the experimental protocol involved rapidly indenting into the sample to a desired depth (10% of the cellular height) [22, 23, 43] and thereon maintaining the depth while monitoring relaxation in the force levels upto a few seconds followed by retracting the tip from the sample. The ramp script process occurs over a 10 second time frame for each datapoint resulting into individual force-relaxation (F-R) curve. For both nanoindentation and ramp script procedures, we incorporated at least 7 cells each with 10 datapoints assigned to nanoindentation and at least 5 datapoints assigned to ramp script procedure for each treatment. Finally, all the experiments were conducted at 37°C maintained using a temperature-controlled AFM stage.

### Contact mechanics model employed for AFM data analysis

Each individual F-S and F-R was passed through a boxcar filter to reduce noise and improve signal strength. It was then baseline corrected to complete the pre-processing of data. The corrected F-S and F-R curves were then subjected to individual mathematical models to extract linear and non-linear NMPs according to previous published literature [22]. We employed Derjaguin-Muller-Toporov (DMT) model to analyze F-S curve given as follows:

(1) $$F=\frac{Ka^{3}}{R}-F_{adh}$$

Where, F is the force from the F-S curve, $F_{adh}$ is the adhesion force, K is the effective Young’s modulus given by,

(2) $$\frac{1}{K}=\frac{3}{4}\left(\frac{1-v^{\prime 2}}{E^{\prime }}\right)+\left(\frac{1-v^{2}}{E}\right)$$

And R is the effective radius given by,

(3) $$R={\left(\frac{1}{R_{1}}+\frac{1}{R_{2}}\right)}^{-1}$$

And a is the contact radius given by,

(4) $$a^{2}=R\delta$$

Where, $v^{\mathrm{\prime 2}}$ and $E^{’}$ are Poisson’s ratio ad modulus of the probe whereas, $v^{2}\;$ and E are that of the sample, respectively. $R_{1}$ and $R_{2}$ are the radius of the probe and sample, respectively.

We also employed poroelasticity model to evaluate non-linear NMPs. Drained Poisson’s ratio (D_P) is evaluated as follows,

(5) $$\frac{F\left(0\right)}{F\left(\infty \right)}=2(1-D\_P)$$

In the F-R experiments, as the force levels reach plateau, the interstitial fluid pressure build up from rapid indentation redistributes in the cell commonly termed as drained condition and given by,

(6) $$F\left(\infty \right)=\left(\frac{8}{3}\right)\left(\frac{1}{1-D\_P}\right)G^{\prime \prime }R^{\frac{1}{2}}\delta^{\frac{3}{2}}$$

$F\left(0\right)$ and $F\left(\infty \right)$ can be extracted from the F-R curve. Diffusion coefficient (D_C) and pore size (P_S) can be further extracted from the poroelasticity model given by,

(7) $$\frac{F\left(t\right)-F\left(\infty \right)}{F\left(0\right)-F(\infty )}=0.491e^{-0.908{\left(\tau \right)}^{0.5}}+0.509e^{-1.679\tau }$$

Where, ‘τ’ is the characteristic poroelastic time required for the force to relax. D_C is evaluated from ‘τ’ as follows,

(8) $$\tau =\left(\frac{D\_C*\tau }{R}\right)$$

P_S is directly linked to D_C as follows,

(9) $$D\_C=\frac{2G^{\prime \prime }\left(1-D_{P}\right)}{1-2D_{P}}P\_S$$

Where, κ is the P_S.

### Statistical significance

Both AFM and mRNA data was normalized with respect to the untreated (control) and further used for analysis and plotting. Origin Pro Lab software was used to plot AFM data. GraphPad Prism software was used to plot qRT-PCR data as well as the correlation matrix. For the AFM datasets, statistical significance was calculated using non-parametric Kruskal Wallis test followed by Bonferroni’s correction as some of the datasets failed to satisfy normality criteria. In case of mRNA expression data, Student’s T-test was performed owing to sample size of datasets. Statistical differences were determined to be significant for P<0.05.

## Results

### Alteration in nanomechanical attributes of Panc-1 cells upon various GNP treatments over varying temporal domain

**Figure 1:**
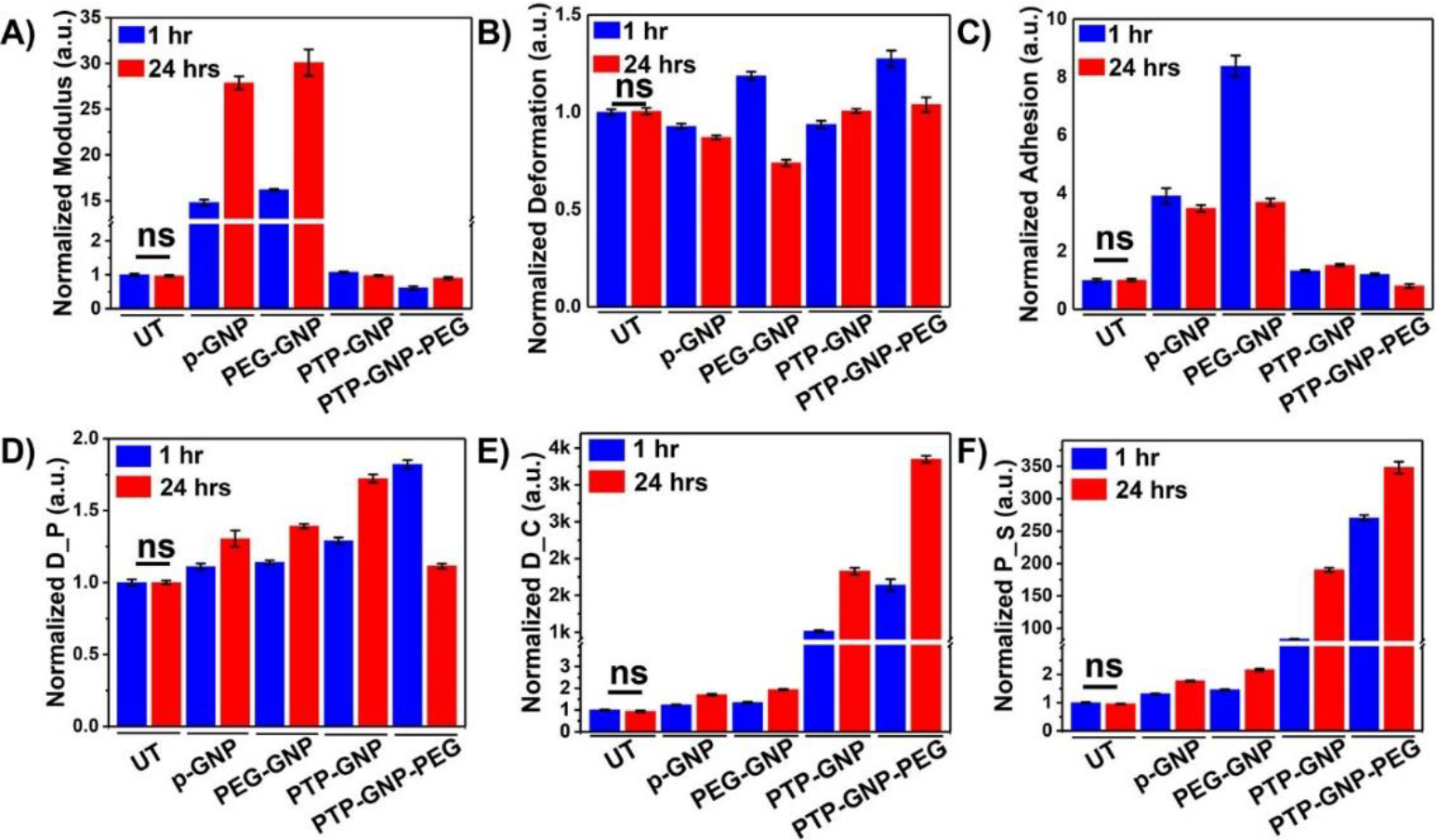
Dynamic alteration in nanomechanical attributes of Panc-1 cells upon various GNP nanoformulation treatments. Normalized linear nanomechanical properties (n=70) such as A) Stiffness, B) Deformation, and C) Adhesion. Normalized non-linear nanomechanical properties (n=35) such as D) D_P, E) D_C, and F) P_S. Statistical significance was performed using One-Way ANNOVA. Significance between 1 and 24 hrs time durations for corresponding treatments was observed to be ****, p<0.0001; unless otherwise mentioned.

Panc-1 cells were treated with various GNP nanoformulations and monitored for their membrane NMPs at the end of 1 hr and 24 hr time points as shown in Figure 1. Linear NMPs such as stiffness, deformation and adhesion were evaluated from the F-S curves, whereas non-linear NMPs such as Drained Poisson’s ratio (D_P), diffusion coefficient (D_C) and pore size (P_S) were evaluated from the F-R curves. Previously, we had studied the short time duration effects of endocytosis on linear and non-linear nanomechanical attributes of PDAC cells. Herein, we evaluate NMPs for longer time durations (upto 24 hr) to incorporate dynamicity of the process. To demonstrate the effectiveness of various treatments, raw data from various GNP treatments was normalized with respect to untreated. Untreated Panc-1 cells demonstrated no significant variations in the linear or non-linear attributes measured over 1 hr and 24 hr time points as shown in Figure 1A and 1D. However, with p-GNP treatment, we observed a ~15-fold increase in membrane stiffness at 1 hr and further ~28-fold increase at 24 hr time points as shown in Figure 1A. Panc-1 cells treated with PEG-GNP demonstrated ~16-fold and ~30-fold increase in their membrane stiffness at 1 and 24 hr time points, respectively. Both these scenarios depict receptor independent endocytosis. During receptor dependent endocytosis of PTP-GNP and PTP-GNP-PEG in Panc-1 cells, we observed minimal increase and decrease in membrane stiffness, respectively, At the end of 1 hr and 24 hr time points in the case of PTP-GNP treatment, we observed ~1.07 fold and 0.97-fold change in membrane stiffness, respectively as shown in Figure 1A. However, PTP-GNP-PEG treatment in Panc-1 cells reduced the membrane stiffness by 0.61-fold and 0.9~fold at 1 and 24 hr time points, respectively. Deformation of Panc-1 cells was observed to vary with various treatments. For instance, p-GNP and PEG-GNP treatments depicting receptor independent process, caused the membrane deformation attribute to reduce except for PEG-GNP at 1 hr time point, where we observed an increase in deformation by 1.18-fold as shown in Figure 1B. p-GNP induced alteration in deformation values corresponding to 0.92-fold and 0.87-fold at 1 hr and 24 hr time points, respectively. PEG-GNP at 24 hr time point reduced the membrane deformation by 0.73-fold. Membrane deformation corresponding to receptor dependent process involving PTP-GNP and PTP-GNP-PEG treatments remained non-significant compared to the untreated except in the case of PTP-GNP-PEG treatment where we observed an increase in membrane deformation by 1.27-fold as shown in Figure 1B. Lastly, adhesion parameter was found to be elevated several folds for receptor independent endocytosis inducing treatments. P-GNP treatments showed an increase by 3.9-fold and 3.47-fold at 1 and 24 hr, respectively while, PEG-GNP showed 8.37-fold and 3.69-fold at 1 hr and 24 hr time points, respectively as shown in Figure 1C.

We then focussed on acquisition of non-linear NMPs (poroelastic attributes) wherein, p-GNP and PEG-GNP treatments systematically increased D_P with time. For instance, p-GNP increased D_P by 1.1-fold and 1.3-fold whereas, PEG-GNP increased D_P by 1.13-fold and 1.4-fold at 1 hr and 24 hr, respectively as shown in Figure 1D. Similarly, treatments inducing receptor dependent endocytosis, PTP-GNP caused 1.28-fold and 1.72-fold increase in D_P at 1 hr and 24 hr, respectively PTP-GNP-PEG treatments at 1 hr and 24 hr with change corresponding to 1.82-fold and 1.18-fold, respectively. D_C was observed to be consistently increase with time durations within a particular treatment irrespective of type of endocytosis as shown in Figure 1E. However, the fold-change in receptor dependent was several thousand folds more compared to the receptor independent endocytosis. P-GNP induced 1.23 and 1.7-fold increase at 1 and 24 hr, respectively whereas, PEG-GNP treatment elevated D_C by 1.34 and 1.93-fold at 1 and 24 hr time points, respectively. PTP-GNP treatments caused an increase by 1014-fold and 1826-fold at 1 and 24 hr time points, whereas PTP-GNP-PEG increased D_C by 1639-fold and 3348-folds, at 1 and 24 hr, respectively as shown in Figure 1E. Finally, P_S followed similar trends in increase compared to D_C. p-GNP treatment caused an increase by 1.31-fold and 1.77-folds at 1 and 24 hr time points, respectively. PEG-GNP also increase P_S by 1.45-fold and 2.16-folds at 1 and 24 hr time points, respectively as shown in Figure 1F. Receptor dependent inducing treatments supported by PTP-GNP treatments in Panc-1 increased P_S size by 83.22-folds and 190-fold corresponding to 1 hr and 24 hr time points, respectively. PTP-GNP-PEG treatment increased P_S by 270-fold and 348-folds at 1 hr and 24 hr time points, respectively as shown in Figure 1F. In conclusion, D_C and P_S were more definitive than other linear and non-linear NMPs that exhibited membrane alterations during receptor dependent and independent endocytosis in Panc-1.

### Alterations in cytokine expressions in Panc-1 cells upon GNP treatments for various durations

**Figure 2:**
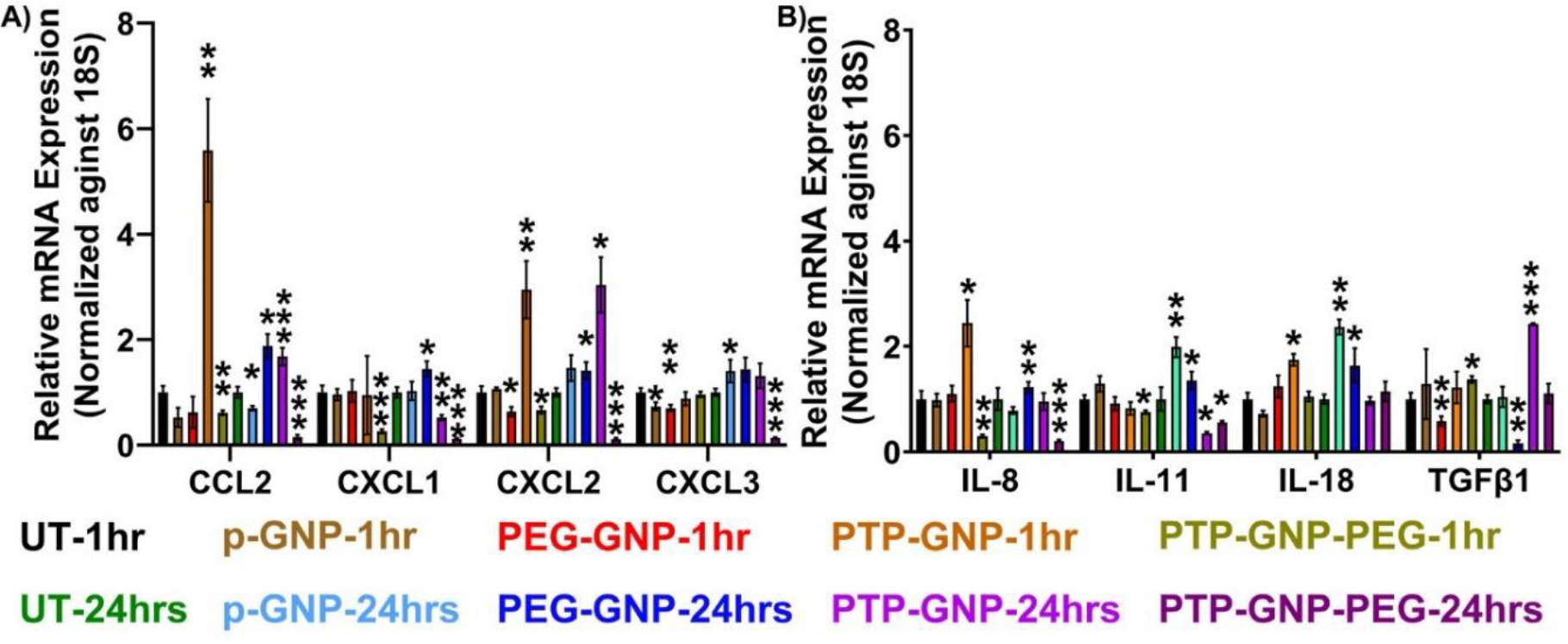
Dynamic alteration in cytokines expressions upon various GNP nanoformulation treatments in Panc-1 cells. A) Chemokines and B) Inflammatory cytokines and growth factor. Statistical significance was performed by T-test as follows, *, p<0.05; **, p<0.01; ***, p<0.001; otherwise, non-significant.

Following experimental treatment regimens from the AFM studies, Panc-1 cells were treated with various GNP formulations for 1 hr and 24 hr and monitored their relative alterations in pro-tumorigenic cytokine expressions. Raw values of respective cytokines were normalized by the untreated value corresponding to the same cytokine expression. In all the cytokines we tested, we did not observe any significant alteration in cytokines expression for Panc-1 at 1 hr and 24 hr duration as seen from Figure 2A and 2B. We observed a 5.7-fold increase in CCL2 expression in the presence of PTP-GNP treatment at 1 hr. We also observed a 2-fold increase in CCL2 expression at 24 hr duration when cells were treated with PTP-GNP and PEG-GNP (Figure 2A). CXCL1 did not show an increase for any treatments. However, we observed a drop in its expression corresponding to PTP-GNP-PEG treatments at 1 and 24 hr with 0.15 and 0.1-fold changes, respectively. In the case of CXCL2, most significant alterations were observed for PTP-GNP treatments at 1 and 24 hr with fold change corresponding to ~3. CXCL3 expression levels remained similar throughout 1 and 24 hr except for PTP-GNP-PEG treatment at 24 hr Figure 2A. IL-8 expression also increased for PTP-GNP at 1 hr Figure 2B. However, we observed a significant decrease in IL-8 expression levels for PTP-GNP-PEG at 1 and 24 hr with fold change corresponding to 0.55 and 0.4, respectively Figure 2B. IL-11 was more sensitive to p-GNP treatment where we observed a 2-fold increase in its expression. We also observed its expression decrease to 0.4-fold and 0.5-fold for PTP-GNP and PTP-GNP-PEG, respectively Figure 2B. IL-18 altered and increased 1.75-fold, 2-fold and 1.6-fold for PTP-GNP at 1 hr, p-GNP at 24 hr and PEG-GNP at 24 hr, respectively. We observed that TGFβ-1 expression varied with various GNP treatments and while it was significantly elevated by 2.4-fold for PTP-GNP at 24 hr, it showed decrease in its expression to 0.5-fold and 0.15-fold for PEG-GNP at 1 hr and 24 hr, respectively Figure 2B. Overall, except for TGFβ-1, we observed a consistent decrease in all the cytokine expressions with PTP-GNP-PEG treatments in Panc-1 cells at 24 hr.

### Correlations between dynamic alterations in nanomechanical attributes and cytokines in Panc-1 cells upon various GNP treatments

**Figure 3:**
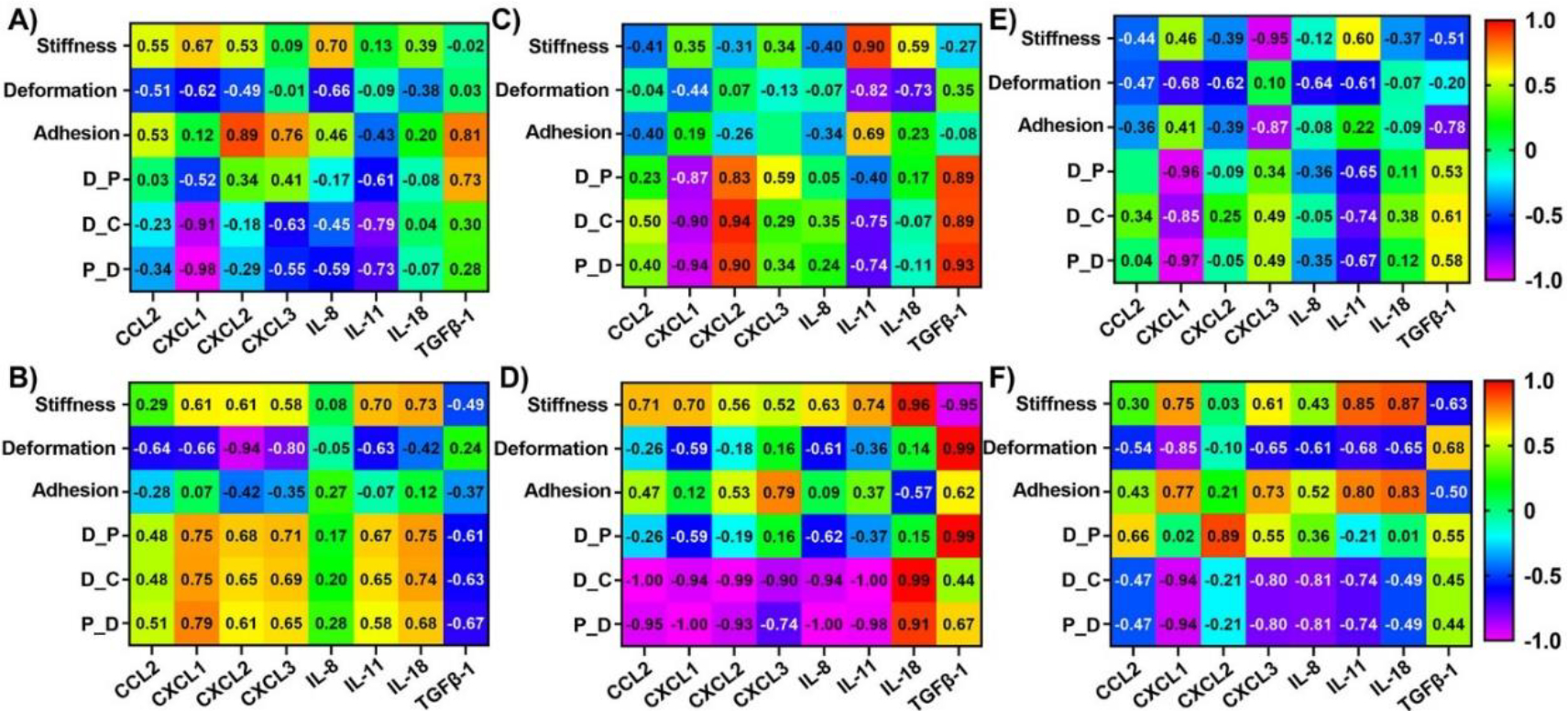
Heat map showing correlation coefficient matrix between nanomechanical attributes and cytokines expressions in Panc-1 cells upon various GNP formulation treatments. Various scenarios explored were A) Receptor dependent, B) Receptor independent, C) No PEGylation, D) PEGylation, E) 1 hr treatment and F) 24 hrs treatment.

To evaluate the influence of nanomechanical attributes on cytokines and vice versa, we calculated correlation coefficients using respective treatments as the common factor. Correlation coefficients were observed ranging from +1 to −1 indicating direct or inverse proportionality, respectively. Correlation coefficients at or above 0.85 were considered as threshold for significance. To get a comprehensive understanding of the cytokines and NMPs, it is imperative to draw comparisons from not only endocytosis type but also surface modifications and time durations. Hence, we further classified these correlations into different categories based on receptor dependent vs independent, PEGylated vs non-PEGylated GNPs and 1 hr vs 24 hr in Figure 3A – 3F. While receptor dependent endocytosis exhibited high correlation of −0.91 and −0.98 for D_C and P_S, respectively with CXCL1 as shown in Figure 3A; receptor independent endocytosis showed high correlation of −0.94 between deformation and CXCL2. Adhesion showed a relatively higher correlation coefficient of 0.89 with respect to CXCL2 expression compared to all other cytokines as shown in Figure 3B. Other NMPs did not exhibit significant correlations with the cytokines panel as indicated by their poor correlation coefficients seen from Figure 3A and 3B. In several instances such as stiffness vs CXCL3, TGFβ-1 as well as deformation vs CXCL3, IL-11 and TGFβ-1, etc. we observed that the correlation coefficient was close to 0, which tells us that these NMPs and corresponding cytokines have no correlation. Then, we observed the influence of PEGylation on these correlations. Stiffness was observed to correlate highly with IL-11 for non-PEGylated GNPs as shown in Figure 3C. We also observed a high correlation between non-linear nanomechanical attributes such as D_P, D_C, P_S and CXCL1, CXCL2 and TGFβ-1 with coefficients above ±0.83 as shown in Figure 3C. These attributes were positively correlated with CXCL2 and TGFβ-1, whereas negatively correlated with CXCL1. In the case of PEGylated GNPs, stiffness was positively and negatively correlated with IL-18 and TGFβ-1 with coefficients at 0.96 and −0.95, respectively as shown in Figure 3D. Deformation and P_S were highly correlated with TGFβ-1 with a coefficient of 0.99. D_C was negatively correlated with coefficients of −0.94 or above for all the cytokines except IL-18 and TGFβ-1. IL-18 and D_C were in fact, positively correlated with coefficient of 0.99. P_S on the other hand was highly negatively correlated with cytokines such as CCL2, CXCL1, CXCL2, IL-8 and IL-11 with coefficients at or above −0.93 as shown in Figure 3D. Similar to D_C, IL-18 was again positively correlated with P_S_ with coefficient of 0.91. Finally, at 1 hr time point, we observed a negative correlation between stiffness and CXCL3 with a coefficient of −0.95 as shown in Figure 3E. Adhesion attribute was negatively correlated with a coefficient of −0.87 with CXCL3, which was significantly higher than other cytokines. All non-linear attributes were negatively correlated with CXCL1 with coefficients at or above −0.85 as shown in Figure 3E. At 24 hr time point, stiffness was observed to be positively correlated with IL-11 and IL-18 with coefficients at 0.85 and 0.87, respectively as shown in Figure 3F. Deformation was observed to be negatively correlated with CXCL1 and the corresponding coefficient was −0.85. D_P was found to be correlated with CXCL2 with a coefficient of 0.89 as shown in Figure 3F. D_C and P_S was observed to be highly negatively correlated with CXCL1 both with a coefficient of −0.94. To sum up, major influence of cytokines on NMPs or vice versa was observed based on surface modification for GNP treatments in Panc-1 cell line. To be precise, the availability or non-availability of PEG coating resulted into higher significant correlations between the NMPs and cytokines. For instance, CXCL1, CXCL2 and TGFβ-1 were all highly correlated with non-linear attributes in Panc-1 cells.

### Alteration in nanomechanical attributes of AsPC-1 cells upon various GNP treatments over varying temporal domain

**Figure 4:**
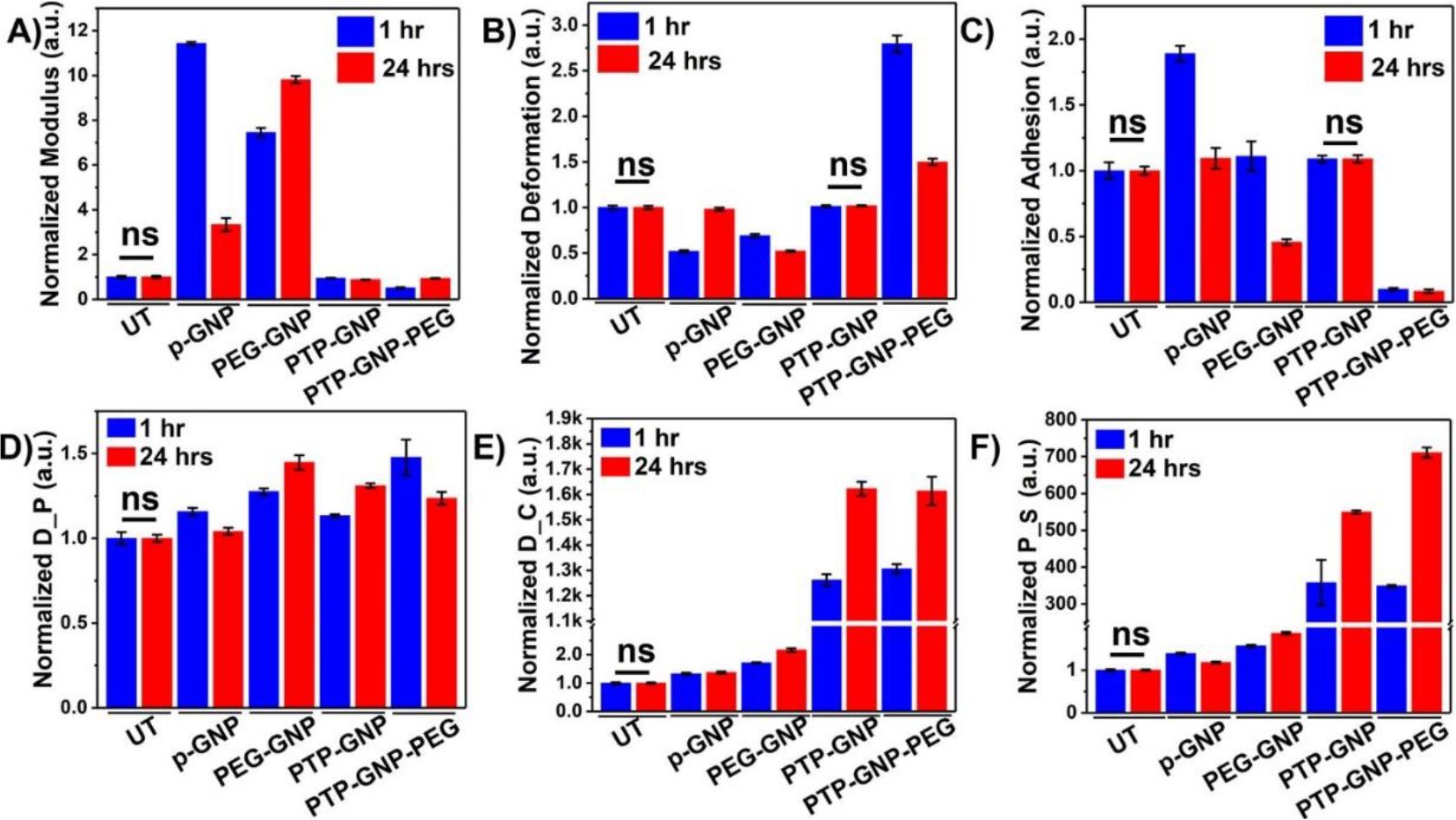
Dynamic alteration in nanomechanical attributes of AsPC-1 cells upon various GNP nanoformulation treatments. Normalized linear nanomechanical properties (n=70) such as A) Stiffness, B) Deformation, and C) Adhesion. Normalized non-linear nanomechanical properties (n=35) such as D) D_P, E) D_C, and F) P_S. Statistical significance was performed using One-Way ANNOVA. Significance between 1 and 24 hrs time durations for corresponding treatments was observed to be ****, p<0.0001; unless otherwise mentioned.

To validate our findings in Panc-1 cells, we incorporated AsPC-1, another PDAC cell line of human origin. We observed non-significant changes in cell membrane linear and non-linear NMPs in untreated cells over 1 and 24 hr time durations. However, various GNP formulations introduced profound alteration in NMPs of cell membrane at these temporal domains. Cell membrane stiffness showed varying trends in its behavior with receptor dependent and independent endocytosis. P-GNP increased the cell membrane stiffness by 11.43-fold and 3.33-fold at 1 and 24 hr time points, respectively as shown in Figure 4A. PEG-GNP systematically increased cell stiffness by 7.45-fold and 9.81-folds at 1 and 24 hr, respectively. PTP-GNP and PTP-GNP-PEG, both caused significant reduction in cell stiffness and the change corresponding to PTP-GNP at 1 and 24 hr was 0.95-fold and 0.88-fold, respectively as shown in Figure 4A. PTP-GNP-PEG induced significant reduction in membrane stiffness by 0.51-fold and 0.94-fold at 1 and 24 hr, respectively as shown in Figure 4A. Deformation attribute for receptor independent endocytosis exhibited a significant reduction by 0.51-fold ad 0.98-fold for p-GNP at 1 and 24 hr, respectively as shown in Figure 4B. PEG-GNP treatment exhibited 0.69-fold and 0.52-fold change at 1 and 24 hr, respectively as shown in Figure 4B. PTP-GNP treatment exhibited non-significant alteration in membrane deformation compared to the untreated for corresponding time durations. PTP-GNP-PEG on the other hand demonstrated an elevated 2.79-fold and 1.5-fold change in deformation at 1 and 24 hr time points, respectively as shown in Figure 4B. Adhesion attribute was observed to be varying with GNP treatments. p-GNP treatment increased cell adhesion at 1 hr by 1.89-fold but increase by only 1.09-fold at 24 hr time as shown in Figure 4C. PEG-GNP showed contrasting change in adhesion. It was observed to increase at 1 hr by 1.1-fold but decrease to 0.45-fold change at 24 hr as shown in Figure 4C. PTP-GNP treatment kept the adhesion attribute unaltered compared to the untreated irrespective of the time. However, PTP-GNP-PEG treatment significantly reduced the adhesion expression to 0.09-fold at 1 hr thereby remaining consistent at 24 hr time point as shown in Figure 4C. Like Panc-1 cells, non-linear NMPs for AsPC-1 cells were more definitive in receptor dependent compared to independent endocytosis. Receptor independent endocytosis inducing treatment such as p-GNP and PEG-GNP altered D_P expression at 1 and 24 hr. At 1 hr, in the presence of p-GNP and PEG-GNP, we observed 1.15-fold and 1.27-fold change whereas, after 24 hr time, we observed D_P to be at 1.04-fold and 1.44-fold for p-GNP and PEG-GNP treatments, respectively as shown in Figure 4D. In the case of receptor dependent endocytosis caused by PTP-GNP, D_P was significantly increase by 1.13-fold and 1.31-fold at 1 and 24 hr time points, respectively. PTP-GNP-PEG treatment caused D_P to increase by 1.47-fold at 1 hr and 1.23-fold at 24 hr time as shown in Figure 4D. D_C was altered by 1.32-fold and 1.37-fold at 1 and 24 hr, respectively when AsPC-1 cells were treated with p-GNP as shown in Figure 4E. In the case of PEG-GNP, we observed a slight increase in fold change compared to p-GNP treatment corresponding to 1.76-fold and 2.16-fold at 1 and 24 hr, respectively as shown in Figure 4E. Several thousand-fold change in D_C was observed in receptor dependent endocytosis. PTP-GNP treatment produced 1261-fold and 1622-fold change at 1 and 24 hr time, respectively, whereas PTP-GNP-PEG altered the D_C by 1306-fold and 1613-fold at 1 and 24 hr time point, respectively as shown in Figure 4E. Lastly, P_S of AsPC-1 cells when treated with p-GNP exhibited 1.39-fold and 1.18-fold change in its value at 1 and 24 hr, respectively as shown in Figure 4F. PEG-GNP on the other hand, induced higher fold changes corresponding to 1.56 and 1.86-fold at 1 and 24 hr time points, respectively. Similar to Panc-1, we observed several hundred-fold changes in P_S during receptor dependent endocytosis caused by PTP-GNP and PTP-GNP-PEG treatments as shown in Figure 4F. We observed 357-fold and 550-fold increase in P_S at 1 and 24 hr, respectively when AsPC-1 was treated with PTP-GNP. Also, PTP-GNP-PEG treatment increase P_S by 348-fold at 1 hr and 711-fold at 24 hr time as shown in Figure 4F. Overall, the effects of receptor dependent endocytosis causing treatments on membrane dynamics in AsPC-1 cells were superior to those in Panc-1 cells. Among the linear attributes, stiffness was observed to be influenced the most (ten-fold times). However, in non-linear NMPs, we observed that D_C and P_S was affected the most by thousand-fold and several hundred-fold, respectively for receptor dependent endocytosis compared to control.

### Alterations in cytokine expressions in AsPC-1 cells upon GNP treatments for various durations

**Figure 5:**
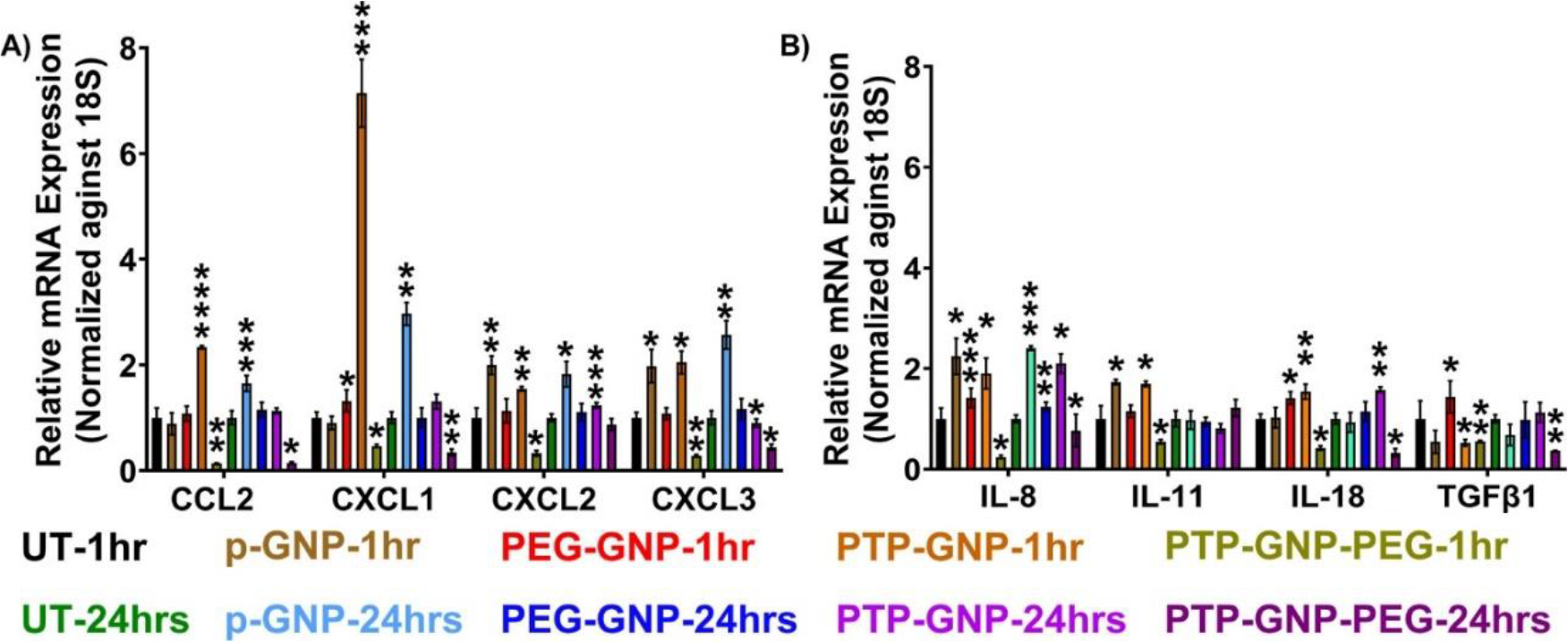
Dynamic alteration in cytokines expressions upon various GNP nanoformulation treatments in AsPC-1 cells. A) Chemokines and B) Inflammatory cytokines and growth factor. Statistical significance was performed by T-test as follows, *, p<0.05; **, p<0.01; ***, p<0.001; ****, p<0.0001; otherwise, non-significant.

We conducted similar study in AsPC-1 in which, we followed the various GNP treatments and time durations along with monitoring the same cytokines as in Panc-1 cells. Here in, we did not observe any significant alteration in untreated cells cytokines at 1 and 24 hr time durations. However, we observed CCL2 to increase by 2.3-fold with PTP-GNP at 1 hr as seen in Figure 5A. Similarly, CXCL1 was elevated 7-fold and 3-fold times with PTP-GNP for 1 hr and p-GNP for 24 hr treatment, respectively. In both CXCL2 and CXCL3, we observed we observed an increase of 2-fold for p-GNP 1 and 24 hr time duration as seen in Figure 5A. IL-8 expression levels were observed to be elevated several folds for all the treatments except PTP-GNP-PEG at 1 hr and 24 hr as seen in Figure 5B. Significant elevation in IL11 expression levels were observed for p-GNP and PTP-GNP at 1 hr corresponding to 1.7-fold change as seen in Figure 5B. Also, IL-18 expression was slightly elevated for PEG-GNP and PTP-GNP at 1 hr with change corresponding to 1.4 and 1.5-folds respectively as seen in Figure 5B. Similar expression levels were also observed for PTP-GNP at 24 hr treatment. Lastly, TGFβ-1 was decreased for most of the treatments in Panc-1 with varying fold changes except for PEG-GNP at 1 hr with an expression increase of 1.4-folds as seen in Figure 5B. Overall, in AsPC-1 cells we observed a consistent decrease in all cytokines including TGFβ-1 for PTP-GNP-PEG 1 and 24 hr. Various GNP formulations in AsPC-1 cells introduced varying pro-tumorigenic cytokine expression levels. Overall, cytokines displayed varying levels of expressions with various GNP treatments. Unlike Panc-1 cell line, this correlation was observed at a significantly diminished level and for fewer criteria in the AsPC-1 cells indicating that genetic variations of the cells play a crucial role during endocytosis.

### Correlations between dynamic alterations in nanomechanical attributes and cytokines in AsPC-1 cells upon various GNP treatments

**Figure 6:**
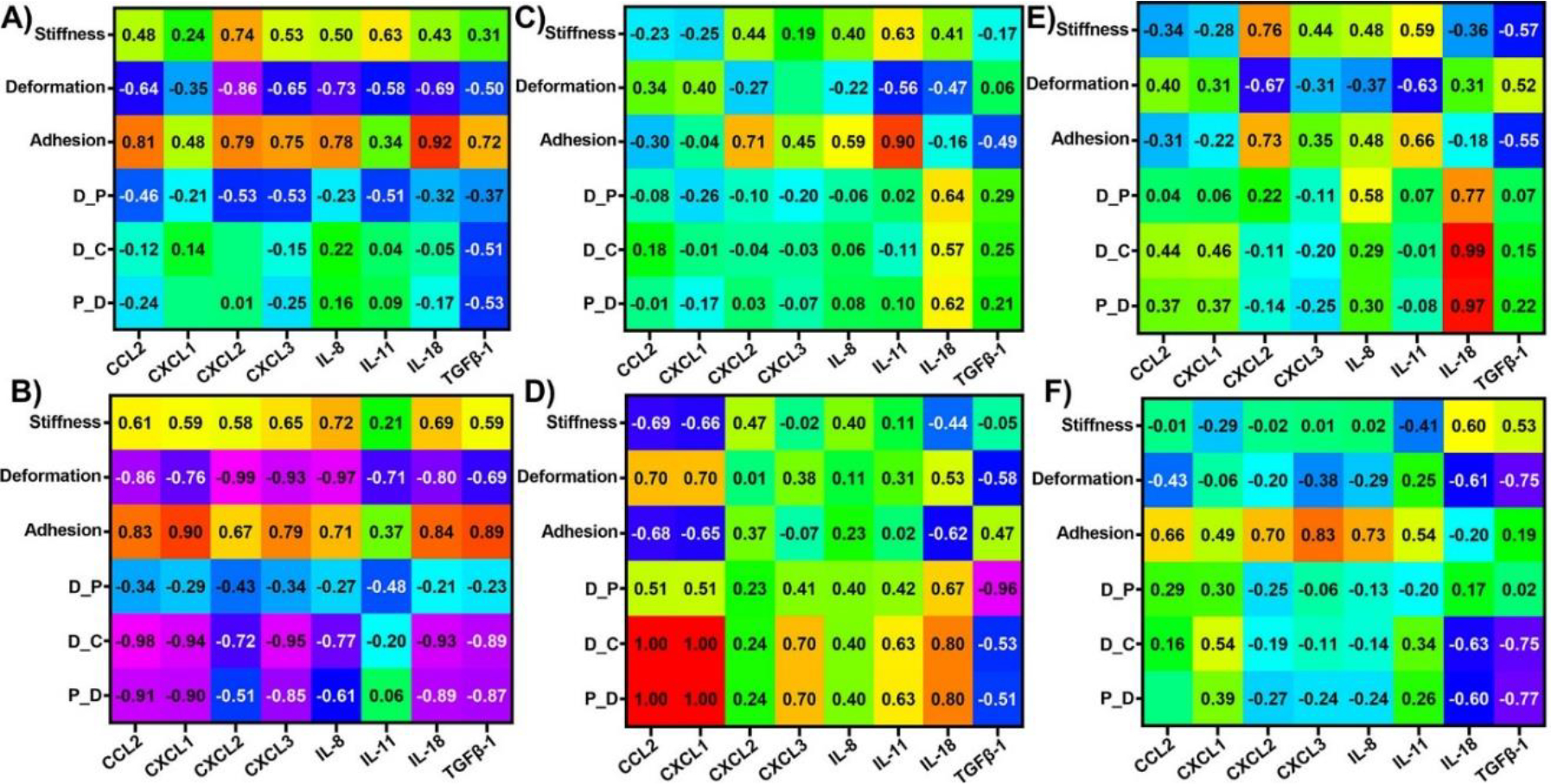
Heat map showing correlation coefficient matrix between nanomechanical attributes and cytokines expressions in AsPC-1 cells upon various GNP formulation treatments. Various scenarios explored were A) Receptor dependent, B) Receptor independent, C) No PEGylation, D) PEGylation, E) 1 hr timepoint, and F) 24 hrs timepoint.

We analyzed the influence of nanomechanical attributes on cytokines expression and vice versa in AsPC-1 cell line. The cut-off criterion was maintained consistent with the Panc-1 cells. In receptor dependent endocytosis criterion, adhesion attribute was observed to be positively correlated with IL-18 with a correlation coefficient of 0.916 as shown in Figure 6A. Whereas, in receptor independent criterion, adhesion was observed to be positively correlated with a coefficient of 0.899 with IL-11 as shown in Figure 6B. Next, we tested the influence of PEGylated GNPs on the nanomechanical attribute and cytokine expressions. When GNPs were not PEGylated such as p-GNP and PTP-GNP, we observed a strong positive correlation between D_C and P_S with IL-18 bearing a correlation coefficient of 0.988 and 0.972, respectively as shown in Figure 6C. More significant correlations were observed for PEGylated GNP treatments such as PEG-GNP and PTP-GNP-PEG. For instance, deformation was strongly negatively correlated with CCL2, CXCL2, CXCL3 and IL-8 with coefficients at −0.86 or above as shown in Figure 6B. Adhesion on the other hand was positively correlated with CXCL1 and TGFβ-1 with coefficients of 0.90 and 0.885, respectively as shown in Figure 6B. D_C was observed to be correlated with CCL2, CXCL1, CXCL3, IL-18 and TGFβ-1 with coefficients at −0.891 or above as shown in Figure 6B. Similarly, P_S was negatively correlated with CCL2, CXCL1, IL-18 and TGFβ-1 with coefficients at or above −0.871 as shown in Figure 6B. At 1 hr time point, we observed a strong positive correlation of 0.996 between CCL2 and both D_C and P_S as well as 0.99 between CXCL1 and both D_C and P_S as shown in Figure 6E. D_P was the other attribute negatively correlated with TGFβ-1 with a coefficient of −0.96 as shown in Figure 6E. We did not observe any significant correlations between nanomechanical attributes and cytokine expression at 24 hr time point in AsPC-1 cells as shown in Figure 6F. Overall, nanomechanical attributes and cytokines were more correlated in Panc-1 cells than AsPC-1 cells under various criteria. The dynamic alterations for different time durations with change in GNP treatment in both cytokines and NMPs affirms the commonly observed and known phenomena that endocytosis is a dynamic process.

## Discussion

Endocytosis is one of the basic phenomena through which, the cell communicates with its surrounding. Endocytosis is pivotal process not only involved in therapeutic nanomedicines reaching their intracellular targets but also for naturally occurring nanoparticles such as viruses and extracellular vesicles [3]. Extensive research on endocytosis by various researchers have significantly improved our understanding yet it is incomprehensive. The influence of size, shape and surface modification of nanoparticles driving the endocytosis process and its consequence on dynamic cellular morphology and nanomechanical alteration are studied [7, 22, 23, 44–47]. Yet, the role of cytokines on NMPs and vice versa during receptor dependent vs independent endocytosis remains to be understudied. To determine the influence of cytokines on NMPs and vice versa, we employed various GNP nanoformulations triggering both receptor dependent and independent endocytosis. Not only the type of endocytosis but also time duration of these treatments and the surface modifications such as PEGylation vs non-PEGylation was evaluated to be a factor in establishing correlations between NMPs and cytokines. We employed two human pancreatic cancer cell line cell lines namely Panc-1, and AsPC-1 and treated them with various GNPs. In our previous publications, we demonstrated that non-linear NMPs such as D_C and P_S are detrimental over linear NMPs in endocytosis. Reports have suggested that stiffness and deformation are complimentary attributes. Stiffer membrane is less prone to deformation [43]. However, through this work we observed a strong correlation between deformation and some of the cytokines such as CCL2, CXCL2, CXCL3 and IL-8 in AsPC-1 cells, but the complimentary nature was not observed to extend to stiffness indicated by poor correlation coefficient. While stiffness, deformation and adhesion are linear NMPs, whereas Drained Poisson’s ratio, diffusion coefficient and pore dimensions are nonlinear NMPs [23]. Linear NMPs rely on F-S curve derived from an instantaneous tip-sample interaction (few milli-seconds). On the other hand, nonlinear NMPs are extracted from the F-R curve, which is the result of longer tip-sample interactions (several seconds) [23]. Non-linear NMPs considers the biphasic nature of cellular entities comprising of both solid-like (store fluid) and liquid-like (dissipate fluid) [23]. Drained Poisson’s ratio gives insights into the correlation between lateral strain and axial strain when cells are under drained condition (after the applied load is removed and the cell relaxes) [48]. Previously, it has been used to distinguish between receptor dependent vs independent endocytosis [23]. It is a condition that has been applied to quantify and image Poisson’s ratio of media [49]. Diffusion coefficient is the measure of cellular membrane’s ability to exchange intracellular and extracellular entities such as cytosolic fluid and nanoparticles among others to pass through the membrane. It is regulated by the cavities in the cell membrane due to various therapeutic treatments as well as endocytosis [50]. One of the studies utilized diffusion coefficient to study the heterogenous nature of lateral diffusion of lipids in Hela cells, which was the result of various microstructures of cytoplasmic membranes [51]. And finally, pore dimensions are the measure of pores in the cell membrane that facilitates free flow of the cytosolic fluid and are often an area of interest [52]. Indeed, various sizes of pores found in the cell membrane facilitate the type of transport mechanisms [53]. Linear NMPs such as stiffness is the ability of the material to withstand the applied load whereas the deformation is how much the sample deforms to the applied load. Adhesion is the measure of repulsive force experienced by the tip as it proceeds with the retraction phase from the sample and not to be confused with adhesion between cell and substrate.

In the past, there have been seldom instances of bridging cytokines and chemokines with nanomechanical attributes [54–56]. For instance, Qi et al., demonstrated that interleukin –1beta (IL-1beta) modulates intracellular cytoskeleton polymerization and regulate cell stiffness [54]. One of the very common CXCL12-CXCR4 pathways was studied to demonstrate that CXCL12-mediated signaling contributed to immune synapse organization thus, influencing T cell activation. CXCR4 downregulation in turn affected actin polymerization and altered microtubule organizing center polymerization. Furthermore, T cell activation was inhibited which, was demonstrated by reduced IL-2 mRNA levels [55]. Articular chondrocyte phenomenon is influenced by cytoskeleton, specifically the actin microfilament architecture. Chondrocytes from superficial zones secrete superficial zone protein (SZP). It was demonstrated by McNary, Sean M. et al., that transforming growth factor β1 (TGFβ1)-induced SZP accumulation was observed when the microtubule cytoskeleton was altered using paclitaxel [56]. Evidence have also demonstrated that cytokines are often modulated during uptake process by the cells [57–59]. For instance, in colorectal cancer, THP-1 monocytes and M0 macrophages uptakes SW480 and SW620-derived EVs efficiently. While SW480-derived EVs increase the CXCL10 expressions in monocytes and M0 macrophage significantly, SW620-derived EVs instigate secretion of IL-6, CXCL10, IL-23 and IL-10 in M0 macrophage [57]. Another study comprised of tumor necrosis factor-α (TNF-α) and Interleukin-1β, which demonstrated that the presence of both these cytokines enhanced endocytosis via the scavenger and mannose receptors in a time-dose dependent in rat liver endothelial cells [58]. In neuroinflammation paradigm, it was demonstrated that mixed glial cells when incubated with pneumococcal lysates from wild-type D39 (serotype 2) pneumococci (D39 wt) enhanced proinflammatory cytokines such as TNF-α and IL-6 as well as the polymorphonuclear chemokine CXCL-2 [59]. Our previous studies coupled with several other literature proves that nanomechanical attributes are key signatures that can differentiate between receptor dependent vs independent process [22]. At the same time, dynamic alteration in the membrane dynamics can be observed during various endocytosis [23]. Moreover, during endocytosis cytoplasmic components such as actin, microtubules rearrange to facilitate endocytosis and in turn influence the nanomechanical attributes of the membrane [60–62]. To sum up, relation between endocytosis and cytokines and nanomechanical attributes have been well studied, yet exact correlations based on endocytosis type and nanomechanical attributes has not been studied to the best of our knowledge, which is demonstrated in the present study. The correlation coefficients convey a relationship between the NMPs and cytokines expression. However, the major unanswered question remains whether the GNP treatments alter nanomechanical attributes, which then causes alteration in cytokine expressions or do the treatments induce cytokine alteration expression that in turn causes alteration in NMPs? Prior results have shown that macrophages treated with silver nanoparticles and/or polystyrene nanoparticles reduced the cell membrane stiffness and negatively correlated with IL-8 expression [63]. Another study explored the antiproliferative and antitumor activities of probiotic Saccaromyces cerevisiae var. boulardii supernatant (SBS) in gastric cancer cell AGS cells. Upon treatment, the authors observed that the cell membrane and adhesion attributes increased and decreased progressively after 24 hr and 48 hr, respectively and were positively and negatively correlated with IL-8 gene expression [64]. As mentioned previously, actin rearrangement is well known to alter membrane stiffness [65, 66]. During the endocytosis, uptake process is accommodated by reorganization in actin [67–69]. In one of the studies, authors observed that by depolymerizing actin using Cytochalasin D caused an up-regulation in IL-8 expression [70]. This study proved that the nanomechanical alteration caused by actin rearrangement led to change in gene expression. A contrasting observation was identified in another study in which, IL-8 and GROα bind in neutrophils to the IL-8α and IL-8β triggering reorganization of actin cytoskeleton in human leukemia cells (HL-60) [71]. In the gastric cancer paradigm, lymph node plays a major role[72]. Authors observed that lymphatic endothelial cells (LECs) and LECs with lymph node metastasis exhibited significant differences in several genes including CXCL1. Further its secretion from LECs stimulated LECs migration, tube formation and F-actin reorganization strengthening the claim that stimulation of cytokines alter nanomechanical attributes in cells [72]. Not only in cancer but also in injury response, chemokines play a crucial role in the recruitment of microglia to areas of central nervous system. Chemokine CCL2 and IL-8 induce migration and alteration in F-actin in adult rat microglia and human microglial cell line, CHME3, *in vitro* [73].

Majority literature points to the fact that in response to external stimulus, various chemokines and inflammatory cytokines induce actin reorganization in turn altering the cellular NMPs. However, the reverse mechanism could still potentially be valid as seen previously [70]. Especially when the cell interacts with the foreign entity foremost and can further lead to cascading of various signaling pathways including the chemokines and inflammatory cytokines response. Ultimately, cause and effect relationships between cytokines and nanomechanical attributes can be heavily dependent on cell types, surface modification of stimulus inducing agents as well as type of endocytosis mechanisms and needs further studies including validations.

## Conclusion

Uptake of gold nanoparticles is associated by reorganization in membrane dynamics leading to alteration in their nanomechanical attribute. The role of various pro-tumorigenic cytokines in endocytosis has been critically demonstrated. However, the exact relation between cytokines and nanomechanical attributes has never been studied for gold nanoparticles endocytosis by pancreatic cancer cell lines. Through our hypothesis we demonstrate that certain pro-tumorigenic cytokines and nanomechanical attributes are correlated on the type of endocytosis and surface chemistry of GNPs containing wide varieties of biologics. The correlations between various linear and non-linear nanomechanical attributes and cytokines revealed that non-linear nanomechanical attributes were highly correlated with most of the cytokines. Moreover, surface modification with PEGylation caused correlations between cytokines and nanomechanical attributes to be more significant than the endocytosis mechanisms or temporal domain studies. Both scenarios in which, cytokines affect nanomechanical attributes and vice versa have been demonstrated previously, we believe that this relation depends on several factors such as specific to cell type and nanoparticles physical and chemical attributes. Future work will involve broadening the cytokine panel to incorporate anti-tumorigenic cytokines and prepare a library comprising of cytokine response and nanomechanical attributes to devise effective therapeutic strategies.

## Supplementary Material

1

## Abbreviations:

GNP: Gold nanoparticles
NMPs: Nanomechanical properties
IL: Interleukin
AFM: Atomic force microscopy
p-GNP: Pristine GNP
PEG-GNP: PEGylated GNP
PTP-GNP: Plectin-1 targeted peptide conjugated with GNP
PTP-GNP-PEG: PTP-GNP coated with PEG
hr: Hour
DMEM: Dulbecco’s Modified Eagle Medium
RPMI: Roswell Park Memorial Institute 1640
HI-FBS: Heat-Inactivated Fetal Bovine Serum
NaOH: Sodium hydroxide
DLS: Dynamic light scattering
F-S: Force-separation
F-R: Force-relaxation
DMT: Derjaguin-Muller-Toporov
D_C: Diffusion coefficient
P_S: Pore size
D_P: Drained Poisson’s ratio
TGFβ1: Transforming growth factor β1
TNF-α: Tumor necrosis factor-α
HL-60: Human leukemia cells
LECs: Lymphatic endothelial cells
