# Supplementary material for: Surface Chemistry of Gold Nanoparticles Modulates Cytokines and Nanomechanical Properties in Pancreatic Cancer Cell Lines: A Correlative Study
Source: Fortune J Health Sci. Author manuscript; Available in PMC 2024 Sep 13. (PMC11065124; doi:10.26502/fjhs.170)
Supplement: 1 [file NIHMS1977541-supplement-1.pdf]

Supplementary Table

Gene	Forward	Reverse
**18s**	GTAACCCGTTGAACCCCATT	CCATCCAATCGGTAGTAGCG
**CCL2**	TTTCCCCTAGCTTTCCCCAG	AACATCCCAGGGGTAGAACTG
**CXCL1**	AACATGCCAGCCACTGTGAT	GCCCCTTTGTTCTAAGCCAG
**CXCL2**	AGATCAATGTGACGGCAGGG	TCTCTGCTCTAACACAGAGGGA
**CXCL3**	AGCTTTCTAGGGACAGCTGGA	CAGTTCCCCACCCTGTCATTTA
**CXCL6**	AGCCTACGCTTCTCCCTGAA	AGGATACCTCCCTCAACAGCA
**IL8/CXCL8**	TGCCAGTGAAACTTCAAGCA	TTGGCCCTTGGCCTCAATTT
**IL11**	CCGTCCTTCCAAAGCCAGAT	GAGACCCAAGAATCCGGGAC
**IL18**	GAGAAGTGTCCCAGGACATGA	GCTAGTCTTCGTTTTGAACAGTGA
**TGFβ1**	ACTATTGCTTCAGCTCCACGG	CCCAAGCGCATCTCGTAGC

Table S1: List of primers used in this study.
